# Análisis de la calidad de una aplicación móvil de inteligencia artificial para la interpretación de ECG

**DOI:** 10.47487/apcyccv.v5i2.363

**Published:** 2024-06-24

**Authors:** Rodrigo Chavez-Ecos, Kiara Camacho-Caballero, Marcelo S. Chavez-Ecos, Miguel A. Chavez-Gutarra, Oscar Aguirre-Zurita, Fabian A. Chavez-Ecos

**Affiliations:** 1 CHANGE Research Working Group, Facultad de Ciencias de la Salud, Carrera de Medicina Humana, Universidad Científica del Sur, Lima, Perú. Universidad Científica del Sur CHANGE Research Working Group Facultad de Ciencias de la Salud, Carrera de Medicina Humana Universidad Científica del Sur Lima Peru; 2 Facultad de Medicina Humana, Universidad Nacional San Luis Gonzaga, Ica, Perú. Universidad Nacional San Luis Gonzaga Facultad de Medicina Humana Universidad Nacional San Luis Gonzaga Ica Peru; 3 Instituto Nacional Cardiovascular «Carlos Alberto Peschiera Carrillo», Departamento de Cardiología, INCOR, Lima, Perú. Instituto Nacional Cardiovascular «Carlos Alberto Peschiera Carrillo» Departamento de Cardiología, INCOR Lima Perú

*Sr. Editor:*

El electrocardiograma (ECG) representa un componente fundamental en el conjunto de herramientas diagnósticas de los médicos en todos los niveles de formación, y desempeña un papel esencial en la evaluación integral del paciente. Aunque su interpretación requiere de experiencia y entrenamiento, se ha demostrado que el porcentaje de precisión en estudiantes de Medicina, residentes, médicos en formación y cardiólogos sin un entrenamiento previo y específico en lectura de ECG, es relativamente bajo (42%; 55,8%; 68,5% y 74,5%, respectivamente). Este porcentaje aumenta tras recibir una formación y entrenamiento adecuado, alcanzando porcentajes de 61,5%; 66,5%; 80,1% y 87,5%, respectivamente ^1^.

La evolución del ECG avanza con la tecnología. Una de las primeras innovaciones fue la introducción de la interpretación computarizada del ECG con el objetivo de mejorar su precisión; sin embargo, no ha logrado proporcionar interpretaciones precisas ^2^. Posteriormente, se desarrolló la inteligencia artificial (IA), utilizando bases de datos más extensas de ECG, obteniendo resultados prometedores en su validación ^3^. Sin embargo, el uso del ECG con IA debe examinarse y validarse cuidadosamente en entornos clínicos y la vida real. Es relevante mencionar que, hasta el momento, estos estudios no han sido realizados en Perú ^4^. A pesar de esta limitación, en el Perú se encuentra disponible una aplicación móvil de IA denominada ECG Reader.

Como consecuencia, los autores evaluaron la calidad del ECG Reader utilizando la escala de valoración de aplicaciones móviles (MARS) compuesta por 19 ítems divididos en 4 escalas objetivas: compromiso (si es entretenido, interesante e interactivo); funcionalidad (si es fácil de usar, navegable y precisa en los botones); estética (diseño gráfico y apariencia visual) y calidad de la información (precisión según los detalles de la descripción de la aplicación, claridad de la información, legibilidad, credibilidad y fundamentación basada en estudios científicos). Además, se incluyó una escala subjetiva para la calificación de cada evaluador, que consideraba aspectos como la frecuencia de uso de la aplicación, la calificación en estrellas y la disposición a pagar por la aplicación. Cada ítem se califica en una escala tipo Likert de 5 puntos (1: bajo - 5: alto). Los autores (RC, MC-E, MC-G y FC) evaluaron dicha aplicación y se obtuvo la media de los resultados ^5^ (Figura 1).

**Figura 1:**
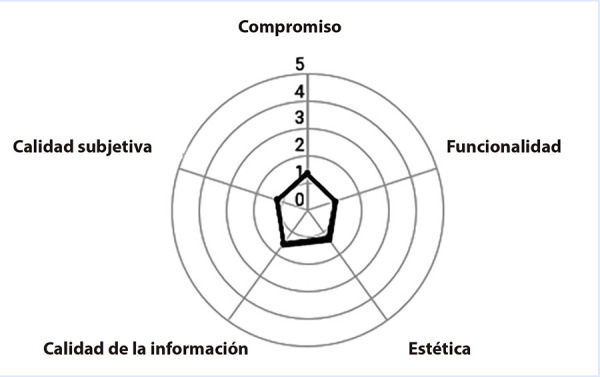
Evaluación usando la herramienta MARS

Asimismo, se identificaron las características de ECG Reader desarrollada por Muhammet Bilgi. Esta aplicación, disponible únicamente en la plataforma iOS desde 2018, tiene un precio de USD 0,99 y no cuenta con una página web disponible. Se encuentra en inglés, tiene un peso de 14,9 MB y es compatible con el iPhone 11 o posterior.

El ECG Reader es solo un ejemplo más de las numerosas aplicaciones móviles de salud que carecen de las características necesarias para ser consideradas de alta calidad y para su implementación en el campo de la salud. Lamentablemente, muchas de estas aplicaciones pasan desapercibidas por las asociaciones u organizaciones encargadas de validar las aplicaciones en el sector sanitario peruano, lo que conlleva al desarrollo y uso de aplicaciones de baja calidad que pueden provocar errores en la toma de decisiones clínicas. De esta misma manera, el estudio de Veazie *et al*. analizó 280 apps relacionadas con el manejo de la diabetes, de las cuales solo 5 (1,8%) cumplían con la concordancia clínica sin alcanzar una alta calidad metodológica ^6^.

En otros contextos sanitarios, como la psiquiatría, el número de apps es mayor y pocas alcanzan una alta calidad ^7^. Enfoques como el Global Digital Health Score, que evalúa aspectos técnicos, clínicos, de usabilidad y de coste, podrían ser una opción para iniciar cuanto antes una regulación estricta de las apps disponibles en nuestro país ^8^.

A pesar de las potenciales contribuciones de la IA para democratizar el cuidado de la salud, es importante destacar que la lectura y la interpretación del ECG a través de las aplicaciones móviles disponibles no debería ser considerada en nuestro medio. Dichas interpretaciones deben ser acompañadas por un profesional de la salud debidamente formado en la lectura de un ECG para garantizar una mayor precisión en su interpretación.

## Financiamiento:

Autofinanciado

## References

[1] Cook DA, Oh SY, Pusic M V (2020). Accuracy of Physicians' Electrocardiogram Interpretations A Systematic Review and Meta-analysis. JAMA Intern Med.

[2] Semigran HL, Levine DM, Nundy S, Mehrotra A (2016). Comparison of Physician and Computer Diagnostic Accuracy. JAMA Intern Med.

[3] Attia ZI, Harmon DM, Behr ER, Friedman PA (2021). Application of artificial intelligence to the electrocardiogram. Eur Heart J.

[4] Shiferaw KB, Wali P, Waltemath D, Zeleke AA (2024). Navigating the AI frontiers in cardiovascular research a bibliometric exploration and topic modeling. Front Cardiovasc Med.

[5] Stoyanov SR, Hides L, Kavanagh DJ, Wilson H (2016). Development and Validation of the User Version of the Mobile Application Rating Scale (uMARS). JMIR Mhealth Uhealth.

[6] Veazie S, Winchell K, Gilbert J, Paynter R, Ivlev I, Eden K, Nussbaum K, Weiskopf N, Guise JM, Helfand M (2018). Mobile Applications for Self-Management of Diabetes.

[7] Steubl LS, Reimann J, Simon L, Terhorst Y, Stach M, Baumeister H (2022). A systematic quality rating of available mobile health apps for borderline personality disorder. Borderline Personal Disord Emot Dysregul.

[8] Mathews SC, McShea MJ, Hanley CL, Ravitz A, Labrique AB, Cohen AB (2019). Digital health a path to validation. NPJ Digit Med.

